# Clinical and laboratory characteristics of symptomatic healthcare workers with suspected COVID-19: a prospective cohort study

**DOI:** 10.1038/s41598-021-93828-y

**Published:** 2021-07-22

**Authors:** Antonin Bal, Karen Brengel-Pesce, Alexandre Gaymard, Grégory Quéromès, Nicolas Guibert, Emilie Frobert, Maude Bouscambert, Mary-Anne Trabaud, Florence Allantaz-Frager, Guy Oriol, Valérie Cheynet, Constance d’Aubarede, Amélie Massardier-Pilonchery, Marlyse Buisson, Julien Lupo, Bruno Pozzetto, Pascal Poignard, Bruno Lina, Jean-Baptiste Fassier, Florence Morfin, Sophie Trouillet-Assant, COVID-SER Study group, Jerôme Adnot, Dulce Alfaiate, Alain Bergeret, André Boibieux, Florent Bonnet, Florence Brunel-Dalmas, Eurydice Caire, Barbara Charbotel, Pierre Chiarello, Laurent Cotte, Constance d’Aubarede, François Durupt, Vanessa Escuret, Pascal Fascia, Juliette Fontaine, Lucie Gaillot-Durand, Myriam Gillet, Matthieu Godinot, François Gueyffier, Laurence Josset, Matthieu Lahousse, Hélène Lozano, Djamila Makhloufi, Marie-Paule Milon, Frédéric Moll, David Narbey, Julie-Anne Nazare, Fatima Oria, Marielle Perry, Virginie Pitiot, Mélanie Prudent, Muriel Rabilloud, Audrey Samperiz, Isabelle Schlienger, Chantal Simon, Martine Valette

**Affiliations:** 1 Laboratoire de Virologie, Institut des Agents Infectieux, Laboratoire associé au Centre National de Référence des virus des infections respiratoires, Hospices Civils de Lyon, Lyon, France; 2 CIRI, Centre International de Recherche en Infectiologie, Team VirPath, Inserm, U1111, Université Claude Bernard Lyon 1, CNRS, UMR5308, ENS de Lyon, 69007 Lyon, France; 3 Joint Research Unit Hospices Civils de Lyon-bioMérieux, Lyon Sud Hospital, Pierre-Bénite, France; 4 Université Claude Bernard Lyon1, Ifsttar, UMRESTTE, UMR T_9405, Lyon University, 8 Avenue Rockefeller, Lyon, France; 5 Occupational Health and Medicine Department, Hospices Civils de Lyon, Lyon, France; 6 Open Innovation and Partnerships (OIP), bioMérieux S.A., 69280 Marcy l’Etoile, France; 7 Institut de Biologie Structurale, CEA, CNRS and Centre Hospitalier Universitaire Grenoble Alpes, Université Grenoble Alpes, Grenoble, France; 8 GIMAP EA 3064 (Groupe Immunité des Muqueuses et Agents Pathogènes), Université Jean Monnet, Lyon University, Saint-Etienne, France; 9 Laboratory of Infectious Agents and Hygiene, University Hospital of Saint-Etienne, Saint-Etienne, France; 10 University Lyon 1, Laboratoire CarMeN, Inserm U1060, INRA U1397, CRNH Rhône-Alpes, Pierre Benite, France; 11 Centre d’appui à la Prévention des Infections Associées aux Soins Auvergne - Rhône-Alpes, Hospices Civils de Lyon - Hôpital H Gabrielle, 20 route de Vourles, Saint Genis Laval, France; 12 UMR5558, Service des Données de Santé, Pôle de Santé Publique, CNRS, Laboratoire de Biométrie et Biologie Evolutive, Hospices Civils de Lyon, Université de Lyon, Université Lyon 1, Villeurbanne, France; 13 Infectious and Tropical Diseases Unit, Hospices Civils de Lyon, Lyon, France; 14 Pôle Santé Publique, Service de Biostatistique Et Bioinformatique ; CNRS, UMR 5558, Laboratoire de Biométrie et Biologie Évolutive, Équipe Biostatistique-Santé, Université de Lyon, Université Lyon 1, Hospices Civils de Lyon, Lyon, France; 15 Department of Dermatology, Hôpital de La Croix-Rousse, Hospices Civils de Lyon, Lyon, France; 16 Department of Anatomy-PathologyCentre Hospitalier Lyon Sud, Hospices Civils de Lyon, Lyon, France; 17 Department of Oral and Maxillofacial Surgery, Hôpital de la Croix-Rousse, Hospices Civils de Lyon, Lyon, France

**Keywords:** Microbiology, Health care

## Abstract

A comprehensive clinical and microbiological assessments of COVID-19 in front-line healthcare workers (HCWs) is needed. Between April 10th and May 28th, 2020, 319 HCWs with acute illness were reviewed. In addition to SARS-CoV-2 RT-PCR screening, a multiplex molecular panel was used for testing other respiratory pathogens. For SARS-CoV-2 positive HCWs, the normalized viral load, viral culture, and virus neutralization assays were performed weekly. For SARS-CoV-2 negative HCWs, SARS-CoV-2 serological testing was performed one month after inclusion. Among the 319 HCWs included, 67 (21.0%) were tested positive for SARS-CoV-2; 65/67 (97.0%) developed mild form of COVID-19. Other respiratory pathogens were found in 6/66 (9.1%) SARS-CoV-2 positive and 47/241 (19.5%) SARS-Cov-2 negative HCWs (*p* = 0.07). The proportion of HCWs with a viral load > 5.0 log_10_ cp/mL (Ct value < 25) was less than 15% at 8 days after symptom onset; 12% of HCWs were positive after 40 days (Ct > 37). More than 90% of cultivable virus had a viral load > 4.5 log_10_ cp/mL (Ct < 26) and were collected within 10 days after symptom onset. Among negative HCWs, 6/190 (3.2%) seroconverted. Our data suggest that the determination of viral load can be used for appreciating the infectiousness of infected HCWs. These data could be helpful for facilitating their return to work.

## Introduction

Since the beginning of the SARS-CoV-2 pandemic in December 2019, healthcare workers (HCWs) from all over the world have been on the front line for the management of COVID-19 patients. Due to close, repeated, and prolonged contacts with COVID-19 patients, HCWs have been a privileged target for SARS-CoV-2 infection^1–3^. Similar to the rest of the population, the clinical spectrum of SARS-CoV-2 infections reported in HCWs encompassed asymptomatic, mild, but also severe and fatal infections^4–7^. The early detection of SARS-CoV-2 infected HCWs is crucial to reduce the risk of nosocomial transmission, which can be associated with an important mortality in at-risk patients^6,8,9^. However, medical resources may not be sufficient to keep infected HCWs on leave for a long time due to HCW shortage. Defining the duration of infectivity of HCWs is therefore of paramount importance for their appropriate management, which becomes crucial to face the current intensive recirculation of the virus in the Northern hemisphere^10,11^.

The diagnosis of SARS-CoV-2 infection is mainly based on RT-PCR performed on naso-pharyngeal swabs (NPS). The virus can be detected about 2 to 3 days before the onset of symptoms and the viral RNA excretion can last up to several weeks depending on the immune competence, the patient age, as well as the severity of the disease^12–17^. As frequently observed for other viral infections, the SARS-CoV-2 viral RNA can be detected beyond the resolution of symptoms, after seroconversion, and without any detectable infectious virus in clinical samples^18–21^. To assess the duration of infectivity of COVID-19 patients, no standard, rapid, and reliable method is available, and consequently the viral isolation in cell culture remains the most appropriate approach despite its fastidiousness^22^. In previous reports, SARS-CoV-2 isolation could be performed up to 10 days after symptom onset in mild-symptomatic patients^18,23–25^ and up to 22 days after the first positive results in severe cases^20,25–27^. To improve the clinical management of SARS-CoV-2 infection in HCWs, a virological investigation including quantitative RT-PCR, viral culture, as well as the determination of neutralizing antibody titers over the course of the disease is needed. With this aim, we performed a comprehensive assessment of COVID-19 in a longitudinal cohort study of 319 front-line HCWs enrolled during the first wave of the pandemic.

## Results

### Demographic and clinical characteristics of SARS-CoV-2 positive and negative HCWs

**Figure 1:**
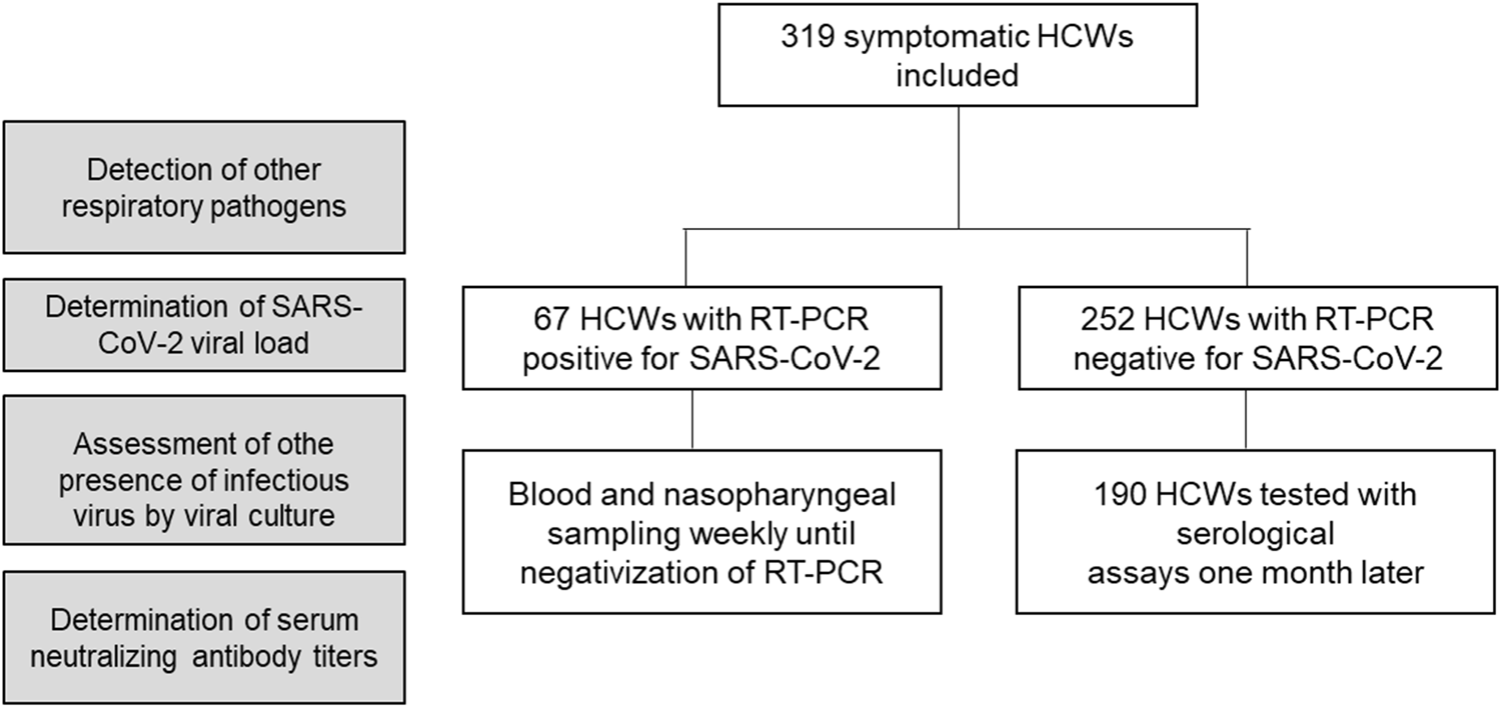
Flow diagram of study population—HCWs, healthcare workers.

Between April 10th and May 28th, 2020, a total of 319 symptomatic HCWs were included (Fig. 1). The main symptoms reported at inclusion were asthenia (260/317, 82%) andheadaches (252/319, 79%). Among the 319 HCWs, 67 (21.0%) were tested positive for SARS-CoV-2. The median [IQR] time between SARS-CoV-2 screening and symptom onset was 3 [2–6] for positive and 5 [3–8] days for negative HCWs (*p* = 0.09). The median age was 36 years old for both positive and negative SARS-CoV-2 HCWs (*p* = 0.93). The sex ratio (M/F) was 1:6 for positive and 1:4 negative participants (*p* = 0.64). The proportion of active smokers was lower among positive SARS-CoV-2 HCWs (6/67, 8.9%) compared to negative SARS-CoV-2 HCWs (64/252, 25.4%; *p*-adjusted = 0.09). The proportion of smell and taste dysfunction was significantly higher in positive participants (26/67, 38.8% for smell and 25/67, 37.3% for taste) than in negative participants (24/250, 9.5% for smell, *p* < 0.001, and 27/250, 10.7% for taste, *p* < 0.001). Diarrhea was reported in 16/67 (23.9%) positive cases and in 104/252 (41.3%) negative cases (*p* = 0.09). Among the 67 positive HCWs, only two severe forms required conventional hospitalization (Table 1), and one of them required ventilation support. No ICU admission was needed.

**Table 1:** Demographic and clinical characteristics of health-care workers (HCWs) exhibiting a negative or a positive RT-PCR test for SARS-CoV-2.

	Negative SARS-CoV-2 HCWs (n = 252)	Positive SARS-CoV-2 HCWs (n = 67)	*p*-value	Adjusted *p*-value
Male sex. n (%)	50 (19.84)	9 (13.43)	0.306	0.638
Age. years. median [IQR]	35.9 [27.5–47.0]	36.0 [29.4–47.7]	0.656	0.926
Body Mass Index*. n	249	67		
median [IQR]	23.3 [21.1–27.1]	24 [22.5–27.3]	0.148	0.473
Currently smoker. n (%)	64/252 (25.4)	6/67 (8.96)	0.013	0.091
Alcohol consumption*. Daily. n(%)	8/251 (3.19)	3/67 (4.48)	0.248	0.606
Presence of comorbidity*. n (%)	109/249 (43.78)	17/66 (25.76)	0.012	0.091
**Description of comorbidity**				
Neurological disorders. n (%)	7 (6.42)	1 (5.88)	1.000	1.000
Cardiovascular disorders. n (%)	2 (1.83)	1 (5.88)	0.355	0.649
Hypertension. n (%)	14 (12.84)	3 (17.65)	0.701	0.935
Heart Failure. n (%)	3 (2.75)	1 (5.88)	0.444	0.735
Diabetes. n (%)	5 (4.59)	0 (0)	1.000	1.000
Immune deficiency. n (%)	2 (1.83)	0 (0)	1.000	1.000
Liver disease. n (%)	2 (1.83)	0 (0)	1.000	1.000
Kidney disease. n (%)	1 (0.92)	1 (5.88)	0.253	0.606
Cancer. n (%)	2 (1.83)	1 (5.88)	0.355	0.649
Hypothyroidy. n (%)	11 (10.09)	2 (11.76)	0.688	0.935
Rheumatic disease. n (%)	2 (1.83)	2 (11.76)	0.088	0.324
Chronic respiratory disease. n (%)	25 (22.94)	1 (5.88)	0.193	0.580
HCW in contact with patients. n (%)	228/252 (90.48)	64/67 (95.52)	0.284	0.634
Delay from symptoms to screening. day. median [IQR]	5 [3–8]	3 [2–6]	0.015	0.091
**Symptom**				
Fever. n (%)	149/252 (59.13)	45/67 (67.16)	0.291	0.634
Sore throat*. n (%)	55/250 (22)	12/67 (17.91)	0.576	0.837
Diarrheas. n (%)	104/252 (41.27)	16/67 (23.88)	0.014	0.091
Pain. n (%)	187/252 (74.21)	53/67 (79.1)	0.505	0.758
Muscular. n (%)	155 (82.89)	50 (94.34)	0.062	0.249
Chest. n (%)	38 (20.32)	11 (20.75)	1.000	1.000
Joints. n (%)	24 (12.83)	3 (5.66)	0.225	0.601
Abdominal. n (%)	72 (38.5)	12 (22.64)	0.048	0.232
Asthenia*. n (%)	204/250 (81.6)	56/67 (83.58)	0.845	1.000
Rhinorrhea *. n (%)	121/250 (48.4)	46/67 (68.66)	0.005	0.079
Nauseas*. n (%)	68/249 (27.31)	14/67 (20.9)	0.365	0.649
Cough. n (%)	135 (53.57)	47 (70.15)	0.022	0.115
Shortness of breath*. n (%)	96/249 (38.55)	21/67 (31.34)	0.346	0.649
Headaches. n (%)	198 (78.57)	54 (80.6)	0.847	1.000
Irritability*. n (%)	65/250 (26)	14/67 (20.9)	0.485	0.758
Anosmia. n (%)	24 (9.52)	26 (38.81)	< 0.001	** < 0.001**
Ageusia. n (%)	27 (10.71)	25 (37.31)	< 0.001	** < 0.001**
Ophthalmic pain. n (%)	28 (11.11)	6 (8.96)	0.775	0.979
Hospitalized. n (%)	0/252 (0)	2/67 (2.98)	0.043	0.187

*Missing data.

### Investigation of bacterial and viral respiratory pathogens

To explore the potential presence of other respiratory infections in symptomatic HCWs tested negative for SARS-CoV-2 and to assess the co-infection rate in COVID-19 HCWs, a multiplex molecular respiratory panel testing was performed. The detection of SARS-CoV-2 with the multiplex panel was fully concordant with the initial routine diagnosis. Other respiratory pathogens were found in 6/66 (9.1%) SARS-CoV-2 positive and 47/241 (19.5%) SARS-Cov-2 negative HCWs (*p* = 0.07). The pathogens responsible for co-infection in the 6 COVID-19 HCWs were rhinovirus/enterovirus (n = 3), adenovirus (n = 2), and parainfluenza virus 2 (n = 2); one patient was tested positive for adenovirus and rhinovirus/enterovirus) (Table 2). The clinical and demographical characteristics of these 6 co-infected patients are detailed in Supplementary Table 1. No bacterial co-infection was found for positive SARS-CoV-2 patients. For negative SARS-CoV-2 HCWs, the most frequent pathogens were rhinovirus/enterovirus (n = 27), adenovirus (n = 15), and other coronaviruses (HKU1 or NL63, n = 7). *Chlamydia pneumoniae* (n = 2) and *Mycoplasma pneumoniae* (n = 1) were also found in negative SARS-CoV-2 HCWs (Table 2).

**Table 2:** Investigation of respiratory pathogens in nasopharyngeal samples from symptomatic healthcare workers (HCWs) using the BioFire Respiratory Panel 2.1 plus.

Respiratory pathogen	Negative SARS-CoV-2 HCWs (n = 241) N (%)	Positive SARS-CoV-2 HCWs (n = 66) N (%)
Coronavirus SARS-CoV-2	0 (0)	66 (100)
Samples with detection of at least one respiratory virus (excluding SARS-CoV-2)	44 (18.26)	6 (9.09)
Adenovirus	15 (6.22)	2 (3.03)
Coronavirus 229E	0 (0)	0 (0)
Coronavirus HKU1	2 (0.83)	0 (0)
Coronavirus NL63	5 (2.07)	0 (0)
Coronavirus OC43	0 (0)	0 (0)
Human metapneumovirus	1 (0.41)	0 (0)
Human rhinovirus/enterovirus	27 (11.20)	3 (4.55)
Influenzavirus A	0 (0)	0 (0)
Influenzavirus B	0 (0)	0 (0)
MERS-CoV	0 (0)	0 (0)
Parainfluenza virus 1	0 (0)	0 (0)
Parainfluenza virus 2	3 (1.24)	2 (3.03)
Parainfluenza virus 3	0 (0)	0 (0)
Parainfluenza virus 4	1 (0.41)	0 (0)
Respiratory syncytial virus	4 (1.66)	0 (0)
Samples with detection of at least one bacterium	3 (1.24)	0 (0)
*Bordetella parapertussis*	0 (0)	0 (0)
*Bordetella pertussis*	0 (0)	0 (0)
*Chlamydia pneumoniae*	2 (0.83)	0 (0)
*Mycoplasma pneumoniae*	1 (0.41)	0 (0)

No significant statistical difference (*p*-value > 0.05) was observed between samples tested negative or positive by SARS-CoV-2 RT-PCR.

### Duration of SARS-CoV-2 PCR positivity according to the normalized viral load

**Figure 2:**
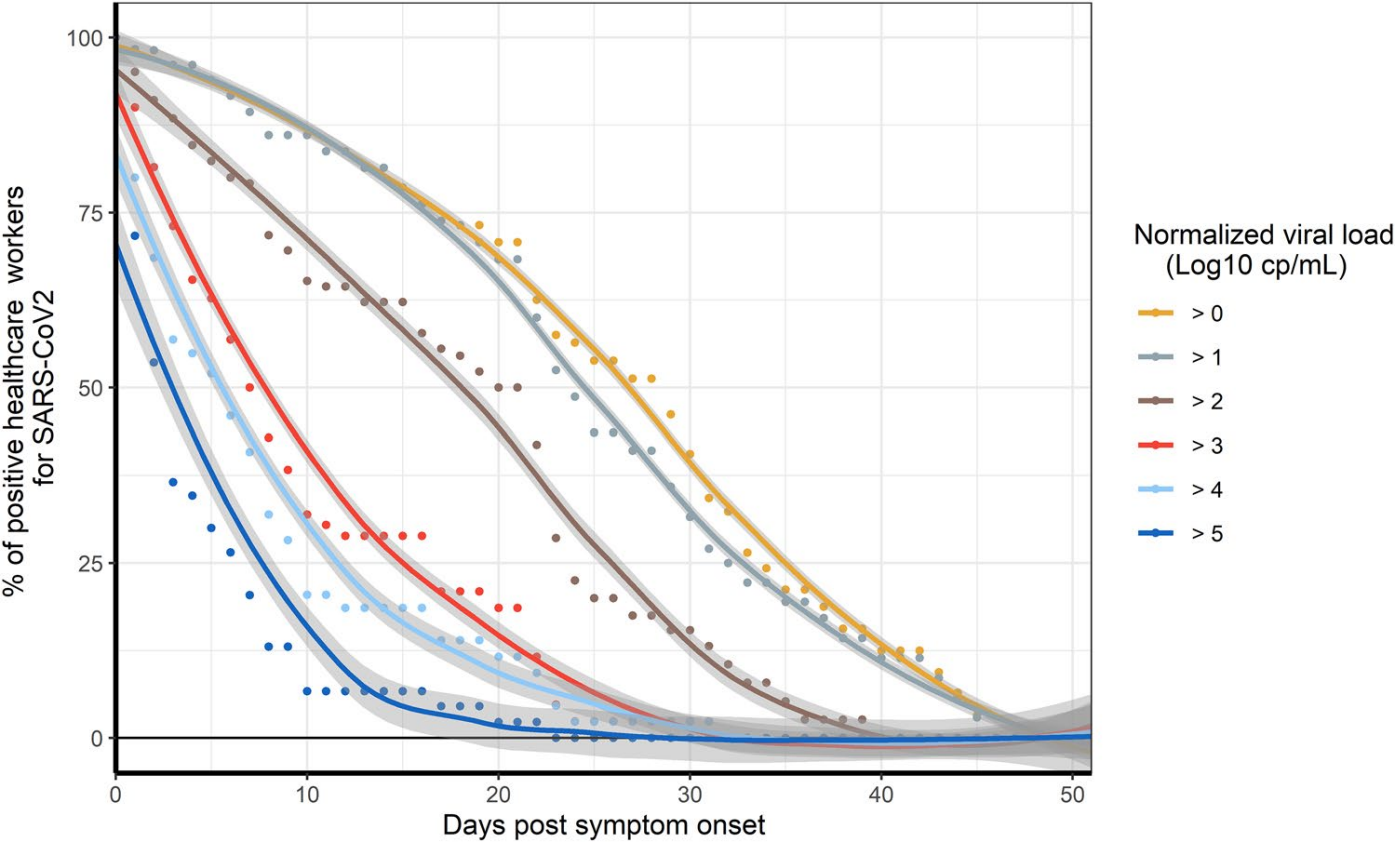
Longitudinal proportion of positive SARS-CoV-2 healthcare workers according to the normalized viral load. Fit Loess curve represents local polynomial regression performed with Loess method. The 95% confidence interval (CI) is indicated (grey area).

For the 61 SARS-CoV-2 positive HCWs without co-infection, the normalized viral load was determined each week until the RT-PCR test was negative (Fig. 2).

The percentage of HCWs with a viral load > 5 log_10_ cp/mL, corresponding to a cycle threshold (Ct) value < 25, rapidly decreased during the first days after symptom onset and reached less than 15% at 8 days. At 20 days after symptom onset, about 40% of HCWs had a positive RT-PCR with a viral load > 2 log_10_ cp/mL. At 40 days post-symptom onset, SARS-CoV-2 RNA was still detectable for 12% of HCWs (< 1 log_10_ cp/mL, Ct value > 37).

### Viral culture results according to the viral load and date of symptom onset

**Figure 3:**
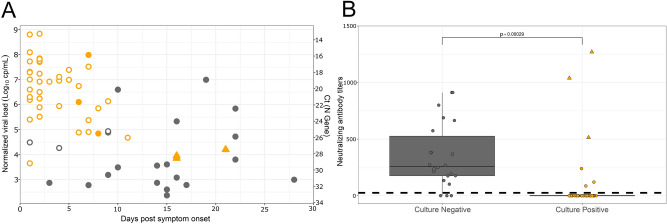
Viral culture results of SARS-CoV-2 according to the post-symptom delay and the presence of neutralizing antibodies. Black circles represent negative virus culture samples and orange circles or triangles represent the positive virus culture samples. Triangles correspond to samples positive in cell culture without cytopathic effect. Solid circles indicate samples with a presence of neutralizing antibodies while empty circles indicate the absence of neutralizing antibodies in serum. (**A**) The Y-axis corresponds to the normalized viral load expressed in Log10 cp/mL or Cycle threshold (Ct) values. (**B**) Viral culture results according to the presence of neutralizing antibodies. Dotted lines correspond to the limit of quantification of neutralizing antibodies.

A total of 64 SARS-CoV-2 RT-PCR positive NPS samples collected from 40 patients without co-infection were inoculated for cell culture (Fig. 3A, Supplementary Fig. 2). Among these, 42/64 (65.6%) samples displayed a positive viral culture. The median [IQR] of the normalized viral load for the cultivable samples was 6.7 [5.6–7.4] log_10_ cp/mL and 3.6 [2.9–4.9] log_10_ cp/mL for non-cultivable samples (*p* < 0.001). The lowest viral load associated with cultivable virus was 3.7 log_10_ cp/mL (Ct value of 30.2; Supplementary Fig. 2, Supplementary Table 2). A total of 38/42 (90%) samples containing cultivable virus had a viral load > 4.5 log_10_ cp/mL, corresponding to a Ct value of 26 for the N gene, and were collected before 10 days after symptom onset.

Further than twelve days after symptom onset, infectious virus could be retrieved in 3 samples, only after subculture. The positivity of these subcultures was established using an RT-PCR test performed on culture supernatant as the Ct difference was > 10 compared to the first passage despite the absence of cytophatic effect (CPE). These 3 samples had a viral load ranging from 3.85 to 4.20 log_10_ cp/mL and were collected from 2 patients (at 15 days after symptom onset for one patient who presented a severe form, and at 16 and 21 days after symptom onset for the other patient who presented a mild form of the disease).

Neutralizing antibody (nAb) titers were measured in contemporaneous serum samples using a SARS-CoV-2 pseudo-typed virus assay. Among individuals with negative viral culture, 18/22 were positive for serum neutralizing activity, suggesting that nAbs may inhibit viral culture from respiratory samples. Of note, 6/7 individuals with a high viral load in NPS (> 4.5 log_10_ cp/mL) and negative viral culture were positive for the detection of nAb. Conversely, high nAb titers were found in the three serum samples of the 2 HCWs with positive viral cultures more than twelve days after symptom onset. We also noticed that 3 additional NPS samples positive for viral culture were from individuals with a contemporaneous nAb activity but at very low titers (Fig. 3B).

### Retrospective SARS-CoV-2 infection assessment with serological testing

Out of the 252 RT-PCR-negative HCWs at initial screening, 190 were serologically tested 1 month after inclusion (V5). Among them, 7/190 were seropositive at V5 with both of the selected serological assays. At inclusion, no other respiratory pathogen was detected using the multiplex respiratory panel for these 7 individuals who developed mild respiratory symptoms (cough (5/7), shortness of breath (5/7), and rhinorrhea (3/7)). Interestingly, 4 of them suffered from ophthalmic pain, and 30 out of the 270 remaining participants tested with the multiplex respiratory panel did.

A potential problem in the sampling quality did not appear to explain the negativity of the SARS-CoV-2 PCR from these 7 seropositive HCWs as the mean cell number in NPS of these individuals (4.0 10^4^ cells/PCR), evaluated using a house-keeping gene, was not significantly different from that of the remaining seronegative HCWs (6.9 10^4^ cells/PCR; *p*-value = 0.14).

At inclusion, no anti-SARS-CoV-2 Ab had been detected for 6 of these 7 HCWs. The single HCW who was already seropositive at inclusion was, however, included later in the present study (at 24 days after symptom onset), which may explain the negativity of the PCR test. For the other 6 HCWs, the median (range) time between symptom onset and inclusion (i.e. RT-PCR screening) was 3 (1–4) days, which was similar for PCR positive patients, suggesting that the negative SARS-CoV-2 RT-PCR cannot be explained by a delayed diagnosis. Taken together, SARS-CoV-2 detection was potentially missed in 6/190 (3.15%) HCWs by RT-PCR performed on nasopharyngeal swabs.

## Discussion

In the present study, investigating symptomatic HCWs, a high prevalence of COVID-19 was found, which is consistent with previous reports on this highly exposed population^7,28–30^. Regarding clinical findings, HCWs presented mostly a mild form of the disease, which can be explained by their relatively young age^31^. We confirmed the important proportion of smell and taste dysfunctions^7,32^, and a low rate of co-infection in SARS-CoV-2 positive patients was found. Higher rates of co-infection have been reported elsewhere, which may be explained by the differences in disease severity and in the timing of testing^33,34^.

A very low proportion of HCWs with a negative RT-PCR test at inclusion seroconverted one month later, suggesting the possibility of a false negative PCR test result at initial screening. Several factors can explain false negative results with RT-PCR, including the low sensitivity of some assays^35,36^, the poor quality of samples, or the inappropriate timing of sampling^37^. However, the Cobas RT-PCR assay used for screening herein has been widely evaluated and no lack of sensitivity has been reported^38^. Furthermore, the quality of samples was checked using a cell control^39^ in order to prevent false negative results due to low cell count. Taken together, our findings confirmed the low rate of false negative RT-PCR results using serology testing^40^, although initial reports have wrongly alerted the scientific community about the poor sensitivity of RT-PCR tests for COVID-19 screening^41^. Due to the design of the present study, it cannot be excluded that some individuals may have developed COVID-19 between inclusion and the serological testing performed one month later for negative patients at inclusion.

Herein, a substantial (12%) part of the cohort was still RT-PCR positive 40 days post-symptom onset, which is consistent with the results from a large study conducted in mild-COVID-19 patients, in which 10% of patients had detectable RNA four weeks after symptom onset^14^. In a meta-analysis, a mean duration of RT-PCR positivity of 17 days has been reported with a maximum of 83 days^42^. It is important to emphasize that RT-PCR tests cannot distinguish between infectious virus and non-infectious RNA, and that RNA detection frequently outlasts the duration of infectivity. In the present study, upper respiratory samples from HCWs with positive RT-PCR 10 days after symptom onset (even those with significant viral loads) were mainly found negative in viral culture.

Contagiousness is dependent on many factors, including the presence of upper respiratory symptoms, but seems unlikely 10 days after symptom onset in case of mild infection.

Our findings are consistent with other studies that have reported a less than 6% probability of cultivating the virus after 10 or 15 days^20,25^. Another large study on 3,790 SARS-CoV-2 positive samples inoculated for viral culture has found that a large majority of cultivable samples were collected during the first week^43^. This delay might be in line with the time required for the elicitation of nAbs, as suggested by the limited number of infectious virus found herein in samples with high viral load obtained after seroconversion, and by the contemporaneous presence of nAbs in the serum of most individuals with negative viral culture. Similarly, in a report on hospitalized patients, serum nAb titers have been independently associated with the absence of detection of infectious SARS-CoV-2. The latter study has also found that some hospitalized patients with a low titer of neutralizing antibodies could still have a positive culture, as also observed herein^20^.

Taken together, the time elapsed since the onset of symptoms is crucial for discontinuing the isolation of HCWs with SARS-CoV-2 infection as underlined by CDC guidelines, which recommend to wait for a period of 10 days along with an improvement of the symptoms (https://www.cdc.gov/coronavirus/2019-ncov/hcp/disposition-hospitalized-patients.html). Of note, a RT-PCR performed 10 days after symptom onset is not recommended for discontinuing isolation as it can be positive while patients are no longer infectious.

A viral load threshold could be useful to assess the presence of infectious virus in clinical samples. Herein, the median viral load of positive culture specimens was 6.67 log_10_ copies/mL. In a study among hospitalized patients, the median viral load was 8.14 log_10_ copies/mL^20^ in samples with infectious virus, while another study reported in children a median viral load of 7.2 log_10_ copies/mL^44^. In addition to age^17^, the observed differences in viral load might be related to the severity of the disease, as higher viral loads are usually noticed in patients with severe forms^45^. Although the viral load can vary depending on the gene targeted by quantitative RT-PCR, a viral load of 5 to 6 log_10_ copies/mL has been suggested as a proxy for the presence of infectious virus^18,22^. In the present study, more than 90% of cultivable virus had a viral load > 4.5 log_10_ cp/mL and were collected within 10 days after symptom onset. This viral load corresponded to a Ct value of 26 with the Argene RT-PCR kit targeting the N gene. For most clinical laboratories that cannot afford true quantitative RT-PCR results, Ct values might be helpful to assess the presence of infectious virus, as previously reported^22–25,27,43^. However, as underlined by Han et al*.*, Ct values are highly dependent on the RT-PCR kit used and can be affected by batch effect or PCR conditions. Therefore, Ct values should be interpreted with caution^46^, especially in taking into account the presence of upper respiratory symptoms and the time following symptom onset.

The present study has several limitations. First, the inclusion period between April and June 2020, corresponded to the second half of the first epidemic wave in France, which led to a limited number of enrolled patients. Furthermore, the national lockdown may have had a substantial impact on the circulation of other respiratory viruses and the rate of co-infection is possibly underestimated. In addition, the date of symptom onset can be difficult to determine accurately. As asymptomatic HCWs were not screened, the prevalence of SARS-CoV-2 infection among HCWs was certainly underestimated in our cohort^4,7,30^. Finally, the results of viral culture must be considered with caution as this method can lack sensitivity, as its performance are highly dependent on the proper collection, transport, and rapidity of inoculation of samples on cells. Despite these limitations, the strength of the present study is to combine a clinical assessment with a comprehensive microbiological investigation in a cohort of COVID-19 patients. In particular, the longitudinal determination of SARS-CoV-2 viral load normalized using the HPRT1 housekeeping gene has not been performed so far. This method might be helpful for preventing misinterpretation related to a poor quality of sampling.

In conclusion, the present study confirms the high prevalence of SARS-CoV-2 infection among symptomatic HCWs who mainly developed mild forms of COVID-19. Our data suggest that the normalized viral load can be useful for appreciating the infectiousness of infected HCWs. These patients are unlikely contagious 10 days after symptom onset, regardless of the viral load at diagnosis.Taken together, these data could be very helpful for defining rules for discontinuing isolation of HCWs and facilitating their safe return to work, which should contribute to reduce the risk of staff shortage.

## Methods

### Study design

A prospective longitudinal cohort study was conducted at the university hospital of Lyon, France (*Hospices Civils de Lyon*, HCL)^47^ including HCWs with symptoms suggesting a SARS-CoV-2 infection (at least one of the following symptoms: fever, respiratory symptoms, headaches, anosmia, ageusia). HCWs with a previous positive SARS-CoV-2 RT-PCR test were excluded. Clinical and microbiological data were collected for all included HCWs. The HCWs with negative SARS-CoV-2 PCR at inclusion came back one month later (V5) for SARS-CoV-2 serology testing (Fig. 1; Supplementary Fig. 1).

### Microbiological investigations

HCWs were tested for SARS-CoV-2 using real-time RT-PCR on NPS (Cobas SARS-CoV-2 Test, Roche, Basel, Switzerland). The NSP used were either in Copan Universal Transport Medium (UTM-RT) or in Cobas PCR medium tube. Patients with a positive RT-PCR result at inclusion (V1) came back weekly for blood sampling during 6 weeks, nasopharyngeal sampling was also performed until negativity was obtained by RT-PCR. Microbiological investigation including determination of viral load, detection of other respiratory pathogens, and assessment of the presence of infectious virus by viral culture was performed on NPS. The detection of respiratory pathogens was performed on 307 NPS collected at inclusion (n = 241 negative patients, n = 66 positive patients) with the BioFire Respiratory 2.1 *plus* Panel (RP2.1*plus*) detecting 23 respiratory pathogens including SARS-CoV-2 (bioMérieux, Lyon, France; Supplementary Fig. 1).

SARS-CoV-2 positive HCWs with a co-infection were removed from the rest of the analysis because symptoms could not be exclusively attributed to SARS-CoV-2.

For COVID-19 HCWs, the SARS-CoV-2 load was determined weekly from inclusion until becoming negative by RT-PCR using SARS-CoV-2 R-gene kit (bioMérieux, Lyon, France).

Nucleic acid extraction was performed from 0.2 mL NPS using NUCLISENS easyMAG and amplification was performed using Biorad CFX96. Quantitative viral load was determined using four internally developed quantification standards targeting the SARS-CoV-2N gene: QS1 to QS4 respectively at 2.5.10^6^, 2.5.10^5^, 2.5.10^4^, 2.5.10^3^ copies/mL of a SARS-CoV-2 DNA standard. These QS were controlled and quantified using the Nanodrop spectrophotometer (ThermoFisher) and Applied Biosystems QuantStudio 3D Digital PCR.

In parallel, NPS were tested using the CELL Control R-GENE kit (amplification of the HPRT1 housekeeping gene) that contains 2 quantification standards QS1 and QS2, at 10^4^ copies/µL (50,000 cells/PCR i.e. 1.25.10^6^ cells/mL in our conditions) and 10^3^ copies/µL (5000 cells/PCR i.e. 1.25.10^5^ cells/mL in our conditions) of DNA standard, respectively, to normalize the viral load according to the sampling quality.

$${\text{Normalized}}\;{\text{viral}}\;{\text{load}}\;\left[ {{\text{Log}}_{{10}} {\text{cp}}/{\text{mL}}} \right] = {\text{Log}}10\left[ {\frac{{{\text{Number}}\;{\text{of}}\;{\text{SARS - CoV - 2}}\;{\text{copies}}\;{\text{per}}\;{\text{mL}}}}{{{\text{Number}}\;{\text{of}}\;{\text{cells}}\;{\text{per}}\;{\text{mL}}}} \times 10^{6} \;{\text{cells}}\;{\text{per}}\;{\text{mL}}} \right]$$

Viral culture was performed following interim biosafety guidelines established by WHO^48^ from NPS in UTM-RT only; guanidine contained in the Cobas PCR medium tube prevented culture assay due to cytotoxic activity. RT-PCR positive NPS (64 NPS collected from 17 patients) were inoculated on confluent Vero cells (ATCC CCL-81) with Eagle’s Minimum Essential Media (EMEM) supplemented with 2% penicillin–streptomycin, 1% L-glutamine, and 2% inactivated fetal bovine serum. Plates were incubated at 33 °C with 5% CO_2_ for 96 h. The cytopathic effects (CPE) were monitored daily; samples were harvested when positive, while negative samples at 96 h underwent subculture on new plates. Culture supernatants were sampled at 2 h post-inoculation, at 96 h, and after an additional 96 h of subculture. RNA from supernatants was extracted using the automated MGISP-960 workstation using MGI Easy Magnetic Beads Virus DNA/RNA Extraction Kit (MGI Tech, Marupe, Latvia), and SARS-CoV-2 detection was performed using TaqPath COVID-19 CE-IVD RT-PCR kit on a QuantStudio 5 System (Applied Biosystems, Thermo Fisher Scientific, Waltham, USA).

### Serological investigations

The presence of anti-SARS CoV-2 antibodies was evaluated on serum samples using the Wantai SARS-CoV-2 Ab ELISA kit (Wantai, Beijing, China), which detects total antibodies, and the VIDAS SARS-COV-2 IgG test (bioMérieux, Lyon, France), according to the manufacturers’ instructions. Positivity was established according to the threshold value recommended by each manufacturer.

Neutralizing antibodies were quantified with a neutralization assay using lentiviral pseudotypes on serum samples. Briefly, gag/pol and luciferase plasmids were co-transfected with a SARS-CoV-2 full length S plasmid in HEK293T cells and pseudoviruses were harvested after 72 h. Serial dilutions of human serum were incubated with pseudoviruses at 37 °C for 1 h, then transferred onto HeLa-ACE2 cells in 96-well plates at 10 000 cells/well (Corning). Plates were incubated at 37 °C for 48 h and HeLa-ACE2 cells were further lysed using 1 × luciferase lysis buffer (Oz Biosciences), at room temperature for 1 h. Luciferase activity was measured by adding luciferase substrate (Oz Biosciences), according to the manufacturer’s instructions. Luciferase intensity was then read using a TECAN luminometer. The results from this assay were expressed as the serum dilution required to reduce infection by 50% (neutralization titer).

### Statistical analysis

The median (interquartile range, IQR) was used to express continuous variables. The difference between groups was assessed using the Student’s T test or Mann–Whitney U test, as appropriate. Categorical variables were expressed as count (percentage) and compared using the Chi-square test or Fisher’s exact test. All statistical analyses were conducted using R (the R foundation, https://www.r-project.org/foundation/, version 3.6.1). Adjusted *p*-values were calculated using the Benjamini & Hochberg method. An adjusted *p*-value < 0.05 was considered statistically significant.

### Ethics

The clinical study registered on ClinicalTrial.gov (NCT04341142) has been fully detailed^47^. Written informed consent was obtained from all participants and approval was obtained from the national review board for biomedical research in April 2020 (*Comité de Protection des Personnes Sud Méditerranée I*, Marseille, France; ID RCB 2020-A00932-37).

### Method statement

All methods were carried out in accordance with relevant guidelines and regulations.

## Supplementary Information

Supplementary Information 1.

Supplementary Information 2.

Supplementary Information 3.

Supplementary Information 4.

## References

[1] Wang J, Zhou M, Liu F (2020). Reasons for healthcare workers becoming infected with novel coronavirus disease 2019 (COVID-19) in China. J. Hosp. Infect..

[2] Xiang B (2020). The impact of novel coronavirus SARS-CoV-2 among healthcare workers in hospitals: An aerial overview. Am. J. Infect. Control.

[3] Report of the WHO-China Joint Mission on Coronavirus Disease 2019 (COVID-19). https://www.who.int/publications-detail-redirect/report-of-the-who-china-joint-mission-on-coronavirus-disease-2019-(covid-19).

[4] Treibel TA (2020). COVID-19: PCR screening of asymptomatic health-care workers at London hospital. Lancet.

[5] Zhan M, Qin Y, Xue X, Zhu S (2020). Death from Covid-19 of 23 Health Care Workers in China. N. Engl. J. Med..

[6] Wang, X. *et al.* Nosocomial outbreak of COVID-19 pneumonia in Wuhan, China. *Eur. Respir. J.* 2020 Jun 4;55(6):2000544. doi: 10.1183/13993003.00544-2020. 10.1183/13993003.00544-2020 PMC7236818 32366488

[7] Gómez-Ochoa SA (2020). COVID-19 in healthcare workers: A living systematic review and meta-analysis of prevalence, risk factors, clinical characteristics, and outcomes. Am. J. Epidemiol..

[8] Quéromès G, Destras G, Bal A, Regue H, Burfin G, Brun S, Fanget R, Morfin F, Valette M, Trouillet-Assant S, Lina B, Frobert E, Josset L. Characterization of SARS-CoV-2 ORF6 deletion variants detected in a nosocomial cluster during routine genomic surveillance, Lyon, France. Emerg Microbes Infect. 2021 Dec;10(1):167-177. doi: 10.1080/22221751.2021.1872351. 10.1080/22221751.2021.1872351 PMC7850418 33399033

[9] Rickman HM (2020). Nosocomial transmission of COVID-19: A retrospective study of 66 hospital-acquired cases in a London teaching hospital. Clin. Infect. Dis..

[10] Zhang JC, Findlater A, Cram P, Adisesh A (2020). Return to work for healthcare workers with confirmed COVID-19 infection. Occup Med (Lond).

[11] Rhee C, Kanjilal S, Baker M, Klompas M (2020). Duration of severe acute respiratory syndrome coronavirus 2 (SARS-CoV-2) infectivity: When is it safe to discontinue isolation?. Clin. Infect. Dis..

[12] Pan Y, Zhang D, Yang P, Poon LLM, Wang Q (2020). Viral load of SARS-CoV-2 in clinical samples. Lancet Infect. Dis..

[13] Weiss A, Jellingsø M, Sommer MOA (2020). Spatial and temporal dynamics of SARS-CoV-2 in COVID-19 patients: A systematic review and meta-analysis. EBioMedicine.

[14] Omar S (2020). Duration of SARS-CoV-2 RNA detection in COVID-19 patients in home isolation, Rhineland-Palatinate, Germany, 2020—An interval-censored survival analysis. Euro Surveill..

[15] He X (2020). Temporal dynamics in viral shedding and transmissibility of COVID-19. Nat. Med..

[16] Wei WE (2020). Presymptomatic transmission of SARS-CoV-2—Singapore, January 23–March 16, 2020. MMWR Morb.Mortal. Wkly Rep..

[17] Benefield, A. E. *et al.* SARS-CoV-2 viral load peaks prior to symptom onset: A systematic review and individual-pooled analysis of coronavirus viral load from 66 studies. *medRxiv* 2020.09.28.20202028 (2020). 10.1101/2020.09.28.20202028.

[18] Wölfel R (2020). Virological assessment of hospitalized patients with COVID-2019. Nature.

[19] Atkinson B, Petersen E (2020). SARS-CoV-2 shedding and infectivity. Lancet.

[20] van Kampen JJA, van de Vijver DAMC, Fraaij PLA, Haagmans BL, Lamers MM, Okba N, van den Akker JPC, Endeman H, Gommers DAMPJ, Cornelissen JJ, Hoek RAS, van der Eerden MM, Hesselink DA, Metselaar HJ, Verbon A, de Steenwinkel JEM, Aron GI, van Gorp ECM, van Boheemen S, Voermans JC, Boucher CAB, Molenkamp R, Koopmans MPG, Geurtsvankessel C, van der Eijk AA. Duration and key determinants of infectious virus shedding in hospitalized patients with coronavirus disease-2019 (COVID-19). Nat Commun. 2021 Jan 11;12(1):267. doi: 10.1038/s41467-020-20568-4. 10.1038/s41467-020-20568-4 PMC7801729 33431879

[21] Walsh KA (2020). SARS-CoV-2 detection, viral load and infectivity over the course of an infection. J. Infect..

[22] Huang C-G (2020). Culture-based virus isolation to evaluate potential infectivity of clinical specimens tested for COVID-19. J. Clin. Microbiol..

[23] Bullard J (2020). Predicting infectious SARS-CoV-2 from diagnostic samples. Clin. Infect. Dis..

[24] La Scola B (2020). Viral RNA load as determined by cell culture as a management tool for discharge of SARS-CoV-2 patients from infectious disease wards. Eur. J. Clin. Microbiol. Infect. Dis..

[25] Singanayagam A, Patel M, Charlett A, Lopez Bernal J, Saliba V, Ellis J, Ladhani S, Zambon M, Gopal R. Duration of infectiousness and correlation with RT-PCR cycle threshold values in cases of COVID-19, England, January to May 2020. Euro Surveill. 2020 Aug;25(32):2001483. doi: 10.2807/1560-7917.ES.2020.25.32.2001483 10.2807/1560-7917.ES.2020.25.32.2001483 PMC7427302 32794447

[26] Liu W-D (2020). Prolonged virus shedding even after seroconversion in a patient with COVID-19. J. Infect..

[27] Gniazdowski V (2020). Repeat COVID-19 molecular testing: Correlation of SARS-CoV-2 culture with molecular assays and cycle thresholds. Clin. Infect. Dis..

[28] Hunter E (2020). First experience of COVID-19 screening of health-care workers in England. Lancet.

[29] Contejean A (2020). Comparing dynamics and determinants of SARS-CoV-2 transmissions among health care workers of adult and pediatric settings in central Paris. Clin. Infect. Dis..

[30] Rivett L, Sridhar S, Sparkes D, Routledge M, Jones NK, Forrest S, Young J, Pereira-Dias J, Hamilton WL, Ferris M, Torok ME, Meredith L; CITIID-NIHR COVID-19 BioResource Collaboration, Curran MD, Fuller S, Chaudhry A, Shaw A, Samworth RJ, Bradley JR, Dougan G, Smith KG, Lehner PJ, Matheson NJ, Wright G, Goodfellow IG, Baker S, Weekes MP. Screening of healthcare workers for SARS-CoV-2 highlights the role of asymptomatic carriage in COVID-19 transmission. Elife. 2020 May 11;9:e58728. doi: 10.7554/eLife.58728. 10.7554/eLife.58728 PMC7314537 32392129

[31] Gomez E, Gustafson DR, Rosenblatt JE, Patel R (2011). Actinobaculum bacteremia: A report of 12 cases. J. Clin. Microbiol..

[32] Maechler F (2020). Epidemiological and clinical characteristics of SARS-CoV-2 infections at a testing site in Berlin, Germany, March and April 2020-a cross-sectional study. Clin. Microbiol. Infect..

[33] Kreitmann L, Monard C, Dauwalder O, Simon M, Argaud L (2020). Early bacterial co-infection in ARDS related to COVID-19. Intensive Care Med..

[34] Kim D, Quinn J, Pinsky B, Shah NH, Brown I (2020). Rates of co-infection between SARS-CoV-2 and other respiratory pathogens. JAMA.

[35] Etievant S, Bal A, Escuret V, Brengel-Pesce K, Bouscambert M, Cheynet V, Generenaz L, Oriol G, Destras G, Billaud G, Josset L, Frobert E, Morfin F, Gaymard A. Performance Assessment of SARS-CoV-2 PCR Assays Developed by WHO Referral Laboratories. J Clin Med. 2020 Jun 16;9(6):1871. doi: 10.3390/jcm9061871. 10.3390/jcm9061871 PMC7355678 32560044

[36] Vogels CBF, Brito AF, Wyllie AL, Fauver JR, Ott IM, Kalinich CC, Petrone ME, Casanovas-Massana A, Catherine Muenker M, Moore AJ, Klein J, Lu P, Lu-Culligan A, Jiang X, Kim DJ, Kudo E, Mao T, Moriyama M, Oh JE, Park A, Silva J, Song E, Takahashi T, Taura M, Tokuyama M, Venkataraman A, Weizman OE, Wong P, Yang Y, Cheemarla NR, White EB, Lapidus S, Earnest R, Geng B, Vijayakumar P, Odio C, Fournier J, Bermejo S, Farhadian S, Dela Cruz CS, Iwasaki A, Ko AI, Landry ML, Foxman EF, Grubaugh ND. Analytical sensitivity and efficiency comparisons of SARS-CoV-2 RT-qPCR primer-probe sets. Nat Microbiol. 2020 Oct;5(10):1299-1305. doi: 10.1038/s41564-020-0761-6. Epub 2020 Jul 10. PMID: 32651556. 10.1038/s41564-020-0761-6 PMC9241364 32651556

[37] Ginocchio CC, McAdam AJ (2011). Current best practices for respiratory virus testing. J. Clin. Microbiol..

[38] Poljak M, Korva M, Knap Gašper N, Fujs Komloš K, Sagadin M, Uršič T, Avšič Županc T, Petrovec M. Clinical Evaluation of the cobas SARS-CoV-2 Test and a Diagnostic Platform Switch during 48 Hours in the Midst of the COVID-19 Pandemic. J Clin Microbiol. 2020 May 26;58(6):e00599-20. doi: 10.1128/JCM.00599-20 10.1128/JCM.00599-20 PMC7269406 32277022

[39] Resa C (2014). Development of an efficient qRT-PCR assay for quality control and cellular quantification of respiratory samples. J. Clin. Virol..

[40] Long Q-X (2020). Antibody responses to SARS-CoV-2 in patients with COVID-19. Nat. Med..

[41] Ai T (2020). Correlation of chest CT and RT-PCR testing for coronavirus disease 2019 (COVID-19) in China: A report of 1014 cases. Radiology.

[42] Cevik M, Tate M, Lloyd O, Maraolo AE, Schafers J, Ho A. SARS-CoV-2, SARS-CoV, and MERS-CoV viral load dynamics, duration of viral shedding, and infectiousness: a systematic review and meta-analysis. Lancet Microbe. 2021 Jan;2(1):e13-e22. doi: 10.1016/S2666-5247(20)30172-5. 10.1016/S2666-5247(20)30172-5 PMC7837230 33521734

[43] Jaafar R (2020). Correlation between 3790 qPCR positives samples and positive cell cultures including 1941 SARS-CoV-2 isolates. Clin. Infect. Dis..

[44] L’Huillier AG, Torriani G, Pigny F, Kaiser L, Eckerle I (2020). Culture-competent SARS-CoV-2 in nasopharynx of symptomatic neonates, children, and adolescents. Emerg. Infect. Dis..

[45] Liu Y (2020). Viral dynamics in mild and severe cases of COVID-19. Lancet Infect. Dis..

[46] Han MS, Byun J-H, Cho Y, Rim JH (2020). RT-PCR for SARS-CoV-2: Quantitative versus qualitative. Lancet Infect. Dis..

[47] Trouillet-Assant S, Albert Vega C, Bal A, Nazare JA, Fascia P, Paul A, Massardier-Pilonchery A, D Aubarede C, Guibert N, Pitiot V, Lahousse M, Boibieux A, Makhloufi D, Simon C, Rabilloud M, Trabaud MA, Gueyffier F, Fassier JB; COVID-SER study group. Assessment of serological techniques for screening patients for COVID-19 (COVID-SER): a prospective, multicentric study. BMJ Open. 2020 Nov 24;10(11):e041268. doi: 10.1136/bmjopen-2020-041268. 10.1136/bmjopen-2020-041268 PMC7688438 33234651

[48] Laboratory biosafety guidance related to coronavirus disease (COVID-19). https://www.who.int/publications-detail-redirect/laboratory-biosafety-guidance-related-to-coronavirus-disease-(covid-19).

