# Publisher Correction: Clinical and laboratory characteristics of symptomatic healthcare workers with suspected COVID-19: a prospective cohort study

**DOI:** 10.1038/s41598-021-99051-z

**Published:** 2021-09-23

**Authors:** Antonin Bal, Karen Brengel-Pesce, Alexandre Gaymard, Grégory Quéromès, Nicolas Guibert, Emilie Frobert, Maude Bouscambert, Mary-Anne Trabaud, Florence Allantaz-Frager, Guy Oriol, Valérie Cheynet, Constance d’Aubarede, Amélie Massardier-Pilonchery, Marlyse Buisson, Julien Lupo, Bruno Pozzetto, Pascal Poignard, Bruno Lina, Jean-Baptiste Fassier, Florence Morfin, Sophie Trouillet-Assant, COVID-SER Study group, Jerôme Adnot, Dulce Alfaiate, Alain Bergeret, André Boibieux, Florent Bonnet, Florence Brunel-Dalmas, Eurydice Caire, Barbara Charbotel, Pierre Chiarello, Laurent Cotte, Constance d’Aubarede, François Durupt, Vanessa Escuret, Pascal Fascia, Juliette Fontaine, Lucie Gaillot-Durand, Myriam Gillet, Matthieu Godinot, François Gueyffier, Laurence Josset, Matthieu Lahousse, Hélène Lozano, Djamila Makhloufi, Marie-Paule Milon, Frédéric Moll, David Narbey, Julie-Anne Nazare, Fatima Oria, Marielle Perry, Virginie Pitiot, Mélanie Prudent, Muriel Rabilloud, Audrey Samperiz, Isabelle Schlienger, Chantal Simon, Martine Valette

**Affiliations:** 1 Laboratoire de Virologie, Institut des Agents Infectieux, Laboratoire associé au Centre National de Référence des virus des infections respiratoires, Hospices Civils de Lyon, Lyon, France; 2 CIRI, Centre International de Recherche en Infectiologie, Team VirPath, Inserm, U1111, Université Claude Bernard Lyon 1, CNRS, UMR5308, ENS de Lyon, 69007 Lyon, France; 3 Joint Research Unit Hospices Civils de Lyon-bioMérieux, Lyon Sud Hospital, Pierre-Bénite, France; 4 Université Claude Bernard Lyon1, Ifsttar, UMRESTTE, UMR T_9405, Lyon University, 8 Avenue Rockefeller, Lyon, France; 5 Occupational Health and Medicine Department, Hospices Civils de Lyon, Lyon, France; 6 Open Innovation and Partnerships (OIP), bioMérieux S.A., 69280 Marcy l’Etoile, France; 7 Institut de Biologie Structurale, CEA, CNRS and Centre Hospitalier Universitaire Grenoble Alpes, Université Grenoble Alpes, Grenoble, France; 8 GIMAP EA 3064 (Groupe Immunité des Muqueuses et Agents Pathogènes), Université Jean Monnet, Lyon University, Saint-Etienne, France; 9 Laboratory of Infectious Agents and Hygiene, University Hospital of Saint-Etienne, Saint-Etienne, France; 10 University Lyon 1, Laboratoire CarMeN, Inserm U1060, INRA U1397, CRNH Rhône-Alpes, Pierre Benite, France; 11 Centre d’appui à la Prévention des Infections Associées aux Soins Auvergne - Rhône-Alpes, Hospices Civils de Lyon - Hôpital H Gabrielle, 20 route de Vourles, Saint Genis Laval, France; 12 UMR5558, Service des Données de Santé, Pôle de Santé Publique, CNRS, Laboratoire de Biométrie et Biologie Evolutive, Hospices Civils de Lyon, Université de Lyon, Université Lyon 1, Villeurbanne, France; 13 Infectious and Tropical Diseases Unit, Hospices Civils de Lyon, Lyon, France; 14 Pôle Santé Publique, Service de Biostatistique Et Bioinformatique ; CNRS, UMR 5558, Laboratoire de Biométrie et Biologie Évolutive, Équipe Biostatistique-Santé, Université de Lyon, Université Lyon 1, Hospices Civils de Lyon, Lyon, France; 15 Department of Dermatology, Hôpital de La Croix-Rousse, Hospices Civils de Lyon, Lyon, France; 16 Department of Anatomy-PathologyCentre Hospitalier Lyon Sud, Hospices Civils de Lyon, Lyon, France; 17 Department of Oral and Maxillofacial Surgery, Hôpital de la Croix-Rousse, Hospices Civils de Lyon, Lyon, France

Correction to: *Scientific Reports* 10.1038/s41598-021-93828-y, published online 22 July 2021

The original version of this Article contained an error in the spelling of the author Emilie Frobert which was incorrectly given as Emile Frobert.

In addition, in the Introduction,

“To improve the clinical management of SARS-CoV-2 infection in HCWs, a virological investigation including quantitative RT-PCR, viral culture, as well as the determination of neutralizing antibodytiters over the course of the disease is needed.”

now reads:

“To improve the clinical management of SARS-CoV-2 infection in HCWs, a virological investigation including quantitative RT-PCR, viral culture, as well as the determination of neutralizing antibody titers over the course of the disease is needed.”

The original Article has been corrected.

