# Supplementary material for: Clinical and laboratory characteristics of symptomatic healthcare workers with suspected COVID-19: a prospective cohort study
Source: Sci Rep. 2021 Jul 22;11:14977. doi: 10.1038/s41598-021-93828-y (PMC8298657; doi:10.1038/s41598-021-93828-y)
Supplement: Supplementary file 1 — Supplementary Information 1. [file 41598_2021_93828_MOESM1_ESM.docx]

**
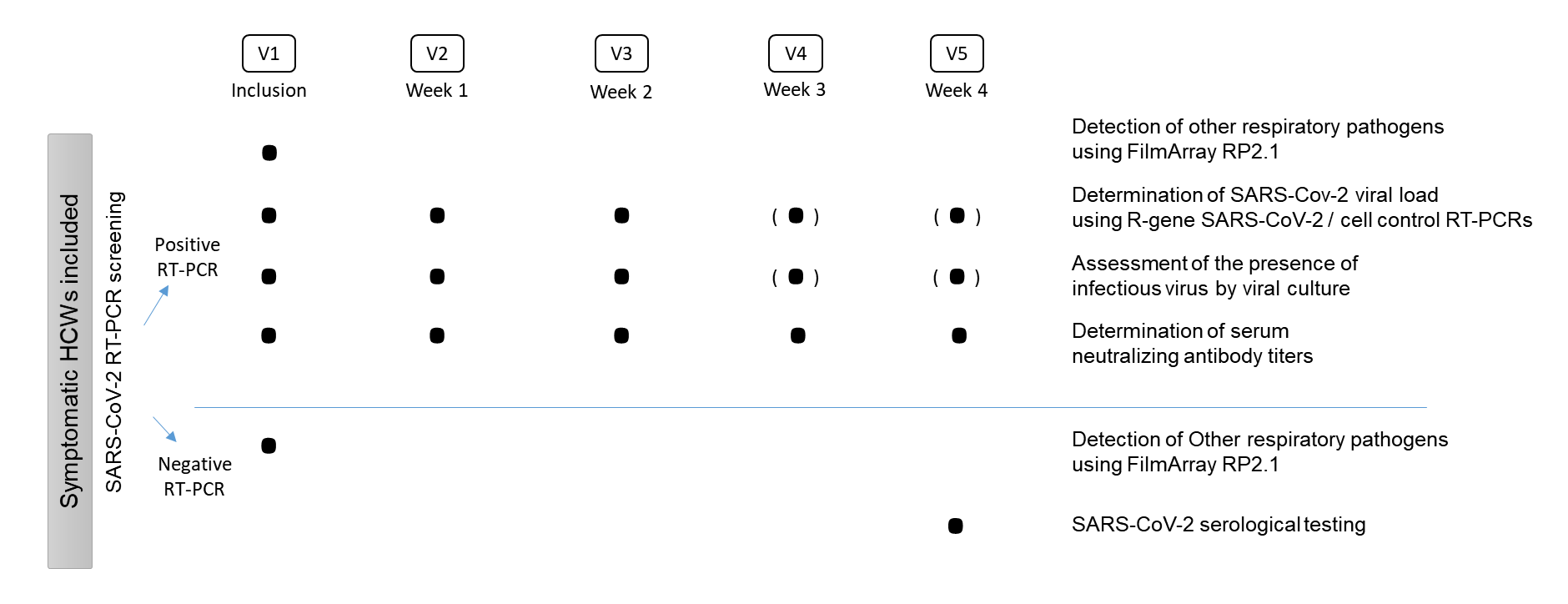
**

**Supplementary Figure 1.** The study design _ Symptomatic HCWs were tested for SARS-CoV-2 using real-time RT-PCR on nasopharyngeal swab (NSP) (Cobas SARS-CoV-2 Test, Roche, Basel, Switzerland). HCWs with negative SARS-CoV-2 PCR at inclusion came back one month later (V5) for SARS-CoV-2 serology testing. Patients with a positive RT-PCR result at inclusion (V1) came back weekly for blood and nasopharyngeal sampling until negativity was obtained by RT-PCR. Brackets indicate that visits take place only if the PCR performed during the previous visit was positive.
