# Supplementary material for: Clinical and laboratory characteristics of symptomatic healthcare workers with suspected COVID-19: a prospective cohort study
Source: Sci Rep. 2021 Jul 22;11:14977. doi: 10.1038/s41598-021-93828-y (PMC8298657; doi:10.1038/s41598-021-93828-y)
Supplement: Supplementary file 2 — Supplementary Information 2. [file 41598_2021_93828_MOESM2_ESM.docx]

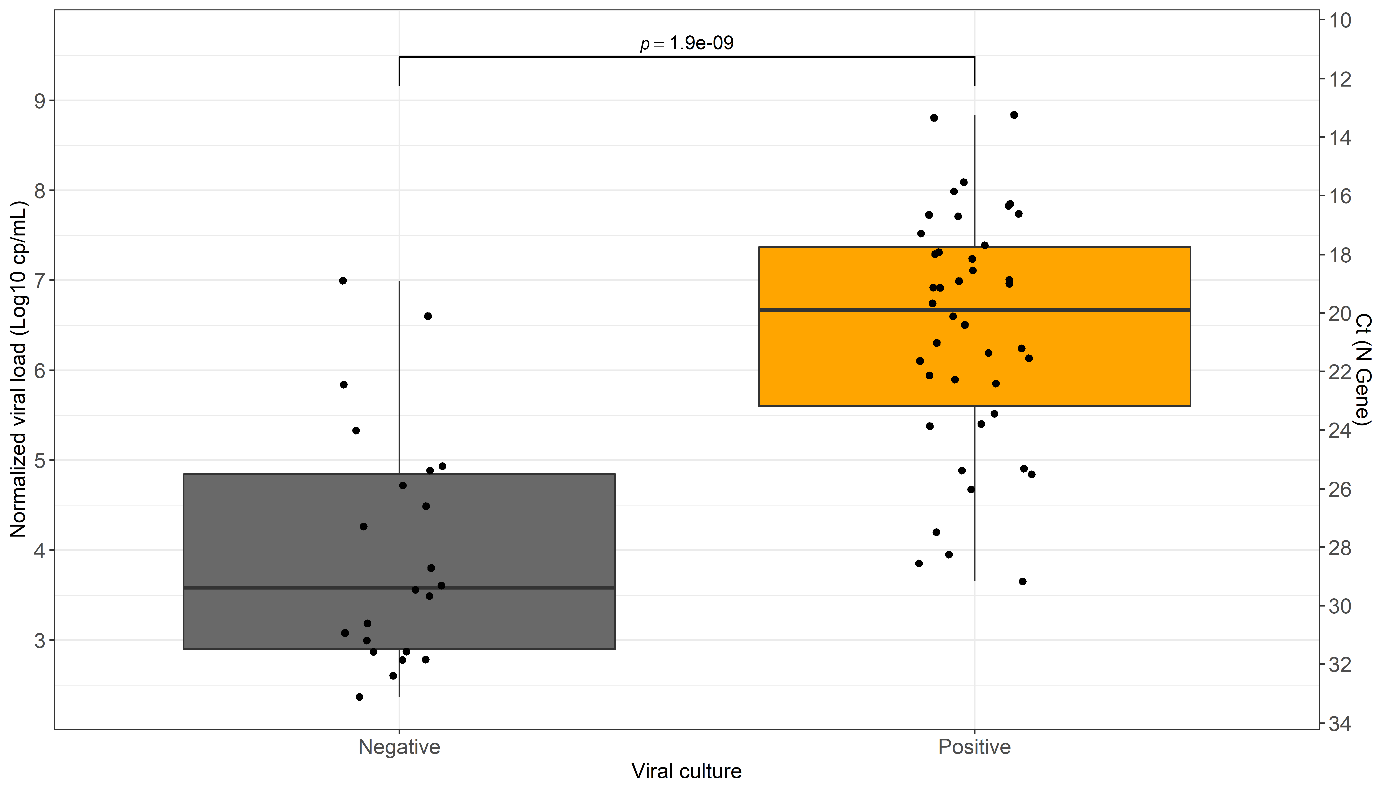


**Supplementary Figure 2.** Normalized SARS-CoV-2 viral load according to the viral culture results. The Y-axis corresponds to the normalized viral load expressed in Log10 cp/mL or Cycle threshold (Ct) values.
