# Supplementary material for: Clinical and laboratory characteristics of symptomatic healthcare workers with suspected COVID-19: a prospective cohort study
Source: Sci Rep. 2021 Jul 22;11:14977. doi: 10.1038/s41598-021-93828-y (PMC8298657; doi:10.1038/s41598-021-93828-y)
Supplement: Supplementary file 3 — Supplementary Information 3. [file 41598_2021_93828_MOESM3_ESM.docx]

|  | Positive SARS-CoV-2 patients (n = 61) | Positive SARS-CoV-2 patients with  co-infection (n=6) | p-value | Adjusted p-value |
| --- | --- | --- | --- | --- |
| Gender. male. n (%) | 8 (13.11) | 1 (16.67) | 1.000 | 1.000 |
| Age. years. median [IQR] | 37.1 [29.4-47.9] | 31.5 [31.1-32.5] | 0.263 | 1.000 |
| Body Mass Index. median [IQR] | 24 [22.5-27.3] | 24.4 [22.8-25.2] | 0.974 | 1.000 |
| Current Smoker. n (%) | 6/61 (9.84) | 0/6 (0) | 0.785 | 1.000 |
| Alcohol consumption. Daily. n (%) | 3/61 (4.92) | 0/6 (0) | 1.000 | 1.000 |
| Presence of Comorbidity*. n (%) | 17/60 (28.33) | 0/6 (0) | 0.326 | 1.000 |
| Description of Comorbidity.  Neurological disorders n (%) | 1 (5.88) | 0 (0) |  |  |
| Cardiovascular disorders n (%) | 1 (5.88) | 0 (0) |  |  |
| Hypertension. n (%) | 3 (17.65) | 0 (0) |  |  |
| Heart Failure n (%) | 1 (5.88) | 0 (0) |  |  |
| Diabetes. n (%) | 0 (0) | 0 (0) |  |  |
| Immune deficiency n (%) | 0 (0) | 0 (0) |  |  |
| Liver disease. n (%) | 0 (0) | 0 (0) |  |  |
| Kidney disease. n (%) | 1 (5.88) | 0 (0) |  |  |
| Cancer. n (%) | 1 (5.88) | 0 (0) |  |  |
| Hypothyroidy. n (%) | 2 (11.76) | 0 (0) |  |  |
| Rheumatic Disease. n (%) | 2 (11.76) | 0 (0) |  |  |
| Chronic respiratory disease n (%) | 1 (5.88) | 0 (0) |  |  |
| HCW Contact patient. n (%) | 59/61 (96.72) | 5/6 (83.33) | 0.249 | 1.000 |
| Post-symptom delay. day. median [IQR] | 3 [2-6.2] | 4 [1.5-5] | 0.893 | 1.000 |
| Ct Value SARSCoV2 N gene**. median [IQR] | 21.3 [17.4-28.2] | 21.5 [15.8-31.8] | 0.903 | 1.000 |
| Symptom. |  |  |  |  |
| Fever. n (%) | 41/61 (67.21) | 4/6 (66.67) | 1.000 | 1.000 |
| Sore throat. n (%) | 12/61 (19.67) | 0/6 (0) | 0.582 | 1.000 |
| Diarrhea. n (%) | 15/61 (24.59) | 1/6 (16.67) | 1.000 | 1.000 |
| Pain. n (%) | 48/61 (78.69) | 5/6 (83.33) | 1.000 | 1.000 |
| Muscular. n (%) | 45 (93.75) | 5 (100) | 1.000 | 1.000 |
| Chest. n (%) | 9 (18.75) | 2 (40) | 0.274 | 1.000 |
| Joints. n (%) | 3 (6.25) | 0 (0) | 1.000 | 1.000 |
| Abdominal. n (%) | 11 (22.92) | 1 (20) | 1.000 | 1.000 |
| Asthenia. n (%) | 51/61 (83.61) | 5/6 (83.33) | 1.000 | 1.000 |
| Rhinorrhea. n (%) | 41 (67.21) | 5 (83.33) | 0.657 | 1.000 |
| Nauseas. n (%) | 14 (22.95) | 0 (0) | 0.330 | 1.000 |
| Cough. n (%) | 44 (72.13) | 3 (50) | 0.353 | 1.000 |
| Shortness of breath. n (%) | 20 (32.79) | 1 (16.67) | 0.657 | 1.000 |
| Headache. n (%) | 49 (80.33) | 5 (83.33) | 1.000 | 1.000 |
| Irritability. n (%) | 12 (19.67) | 2 (33.33) | 0.597 | 1.000 |
| Anosmia. n (%) | 24 (39.34) | 2 (33.33) | 1.000 | 1.000 |
| Ageusia. n (%) | 24 (39.34) | 1 (16.67) | 0.399 | 1.000 |
| Ophthalmic pain. n (%) | 5 (8.2) | 1 (16.67) | 0.444 | 1.000 |
| Hospitalized. n (%) | 2/61 (3.85) | 0/6 (0) | 1.000 | 1.000 |

**Supplementary Table 1** . Demographic and clinical characteristics of SARS-CoV-2 positive health-care workers exhibiting a negative or a positive detection of at least one other respiratory pathogen; * missing data; ** Ct value was determined using SARS-CoV-2 R-gene kit (bioMérieux, Lyon, France).
