# Supplementary material for: Clinical and laboratory characteristics of symptomatic healthcare workers with suspected COVID-19: a prospective cohort study
Source: Sci Rep. 2021 Jul 22;11:14977. doi: 10.1038/s41598-021-93828-y (PMC8298657; doi:10.1038/s41598-021-93828-y)
Supplement: Supplementary file 4 — Supplementary Information 4. [file 41598_2021_93828_MOESM4_ESM.docx]

|  |  | Ct value ( N gene) | | | Log10 cp/mL | | |
| --- | --- | --- | --- | --- | --- | --- | --- |
|  | sample (n) | Median [IQR] | Highest | lowest | Median [IQR] | Highest | lowest |
| Culturable | n=42 | 19.46 [17.31-23.33] | 30.2 | 14.15 | 6.67 [5.6-7.37] | 8.84 | 3.65 |
| Non culturable | n=22 | 29.63 [25.77-30.99] | 34.48 | 18.61 | 3.58 [2.37-4.85] | 7 | 2.37 |

**Supplementary Table 2.** Normalized viral load and Ct-values according to viral culture results.
